# Water-Column Zone Impacts Non-Essential Heavy Metal Accumulation in Fish Occupying Different Zones

**DOI:** 10.3390/toxics13060419

**Published:** 2025-05-22

**Authors:** Meredith Foley, Nesime Askin, Michael Belanger, Carin Wittnich

**Affiliations:** 1 Department of Physiology, University of Toronto, Toronto, ON M5S1A8, Canada; meredithfoley@outlook.com (M.F.); nesimeaskin@yahoo.ca (N.A.); oersdo@gmail.com (M.B.); 2 Oceanographic Environmental Research Society, Barrie, ON L4N2R2, Canada; 3 Department of Surgery, University of Toronto, Toronto, ON M5T1P5, Canada

**Keywords:** ecophysiology, water column, heavy metals, fish

## Abstract

The Gulf of Maine and Bay of Fundy have documented increases in heavy metals with specific properties, resulting in differing concentrations throughout the water column. Whether this impacts metal accumulation in fish that occupy different zones of the water column is unknown; as such, this was the focus of this work. Commercially harvested fish spanning the epipelagic, mesopelagic, and demersal zones of the water column had weight for length (LWR) recorded and biopsies taken. Demersal fish had the highest levels of arsenic and nickel, whereas fish in the epipelagic and demersal zones showed the highest levels of cadmium, lead, mercury and thallium. Compared to historic data, LWR was reduced in one epipelagic species (15%) and two demersal species (24%, 25%). Mesopelagic species showed increased LWR (23%) concurrent with overall lowest metal exposure. These findings demonstrate that fish accumulate non-essential metals at levels dependent on location in the water column, impacting their growth.

## 1. Introduction

Both climate change and anthropogenic sources contribute to heavy metal contamination of the environment and consequently all flora and fauna that occupy it. The world’s marine environment is quite susceptible to this, with both atmospheric and runoff contributing. This results in both surface water contamination and deposition eventually into sediment, which then can be stored for many years and re-released [1]. Fish in this environment are exposed to these in a variety of ways including directly from water through skin and gills, or diet affected by biomagnification dependent on their trophic level, to name two of the most relevant [1]. Interestingly, in contrast, a recent review highlighted that not all heavy metals biomagnify and some may even biodilute, with concentrations decreasing at higher trophic levels, illustrating the complexity of this issue [2]. In addition, where fish reside in the water column may contribute to their contaminant exposure. Understanding the potential impact of these variables on fish levels of contaminants such as non-essential heavy metals is critical, as these are deemed highly toxic at even low levels and negatively affecting growth and weight for length [1]. How species that are commercially harvested for human consumption are affected by such contamination is even more important both from a sustainable biomass perspective as well as a human health perspective, regarding consumption.

The Gulf of Maine (maximum depth 1200 ft.) and the Bay of Fundy (BOF) (maximum depth 250 ft.) are located on the Atlantic coast of the US and Canada [3,4]. The BOF, a world heritage site, is an ecologically critical body of water serving as both a nursery and food source for a multitude of marine species [5]. Two reports have identified increases in contaminants in both the Gulf of Maine and the BOF, specifically in heavy metals (e.g., cobalt, zinc, nickel, copper, arsenic, cadmium, mercury, lead), which raises concerns over the general health of this aquatic ecosystem [6,7]. The most prevalent metals noted at concerning levels (mg/kg) in the sediment of the BOF/GOM near Maine, New Brunswick, and Nova Scotia are arsenic (8.0), cadmium (0.22), lead (20.0), mercury (30.0), nickel (15.0), and thallium (0.3) [8,9,10,11]. Despite earlier legislation to protect these waters (1999 Canadian Environmental Protection Act, 1972 U.S EPA Clean Water Act) [12,13], the prolonged half-life of metals leads to long lasting effects, even decades after metals have been discharged into surrounding waters [14]. Moreover, heavy metals have varying solubility in water, and studies have suggested that metals including arsenic, cadmium, mercury, nickel, and thallium tend to aggregate in the sediment of the ocean floor, leading to speculation that mid to deeper-residing species (demersal zone) should have the highest exposure [15]. However, it has also been documented that metals such as cadmium, lead, and copper can also be found in high concentrations at the top of the water column (epipelagic zone), especially in areas with metals from anthropogenic runoff [16,17]. It should be noted that these metals are unique in that they remain at the surface after initial discharge, but after time will be deposited in the sediment [18]. Despite the unique and extreme tidal patterns, particularly in the BOF, which could potentially increase the resuspension of heavy metals from sequestration, stratification has also been confirmed in this region. Specifically, certain metals have been found in higher concentrations either in the finer sediment in deeper waters or at the surface, and whether this impacts species in the various water-column zones differently is unknown [17,19]. To address this, various commercially available fish species from this region that reside in differing zones were examined for heavy metal accumulation, and since heavy metals can negatively impact growth, possible changes in growth profiles compared to historic controls were also examined.

## 2. Materials and Methods

To assess the potential impact of the different zones of water column in the BOF/GOM, various species, known to primarily occupy specific zones, were collected as follows: (a) epipelagic zone: Atlantic mackerel, *Scomber scombrus* (n = 25) and Atlantic herring, *Clupea harengus* (n = 5); (b) mesopelagic zone: Atlantic pollock, *Pollachius pollachius* (n = 15); and (c) demersal zone: Atlantic cod, *Gadus morhua* (n = 16); Atlantic haddock, *Melanogrammus aeglefinus* (n = 16), and Acadian redfish, *Sebastes fasciatus* (n = 16). Table 1 details sample size, fish trophic level, and their similar respective diets. All fish were commercially harvested and procured from the fishers from the Bay of Fundy and Gulf of Maine during the summers of 2015–2017 (Table 1).

For species with multiple catch locations between BOF/GOM, statistical comparisons confirmed no significant impact of location on the data collected, and therefore were grouped. External examinations and the recording of morphometric assessments (body weight and length) to document weight for length relationships (LWR). Tissue biopsies of muscle and liver were collected for full non-essential heavy metal profiles. The profile analysis was carried out at an accredited government-certified laboratory (University of Guelph Animal Health Laboratory) using an Agilent 7900 Inductively Coupled Plasma Mass Spectrometer (ICPMS-Agilent Technologies, Tokyo, Japan) based on US EPA Method 6020 Inductively Coupled Plasma Mass Spectrometry [20] and a standard method which includes a method blank, control reference, certified reference material, and a duplicate of each sample matrix in each digestion batch. Using certified reference materials, the recovery rate for each element was arsenic 93%, cadmium 100%, lead 101%, mercury 117%, and nickel 89%. Two elements were less than MDL and for these spike recovery was used (antimony 96% and thallium 93%). As a short descriptor, samples were weighed on an analytical balance and placed into 75mL Teflon digestion vessels. Samples were acidified using concentrated nitric acid, hydrochloric acid, and gold, then microwave digested using a CEM corporation (Matthews, NC, USA) MARS Xpress microwave digestion unit. The microwave digest was rinsed with ultrapure filtered water to a final volume of 50mL, after which heavy metals were quantified using ICPMS. Table S1 includes the MDL of the metals assayed, and all values are reported in µg/g wet weight.

Otoliths were collected for aging [21]. The following age ranges were noted (years): mackerel (2–10 years), herring (4–7 years), pollock (2–8 years), cod (2–4 years), haddock (3–6 years), and Acadian redfish (7–20 years). Within these adult age ranges, all fish selected are known to have reached sexual maturity and established themselves in their respective water-column zones, appreciating that they will transiently pass through other zones. These relatively small age ranges, which are approximately 10% of their lifespan, did not appear to influence the heavy metal data collected (e.g., some r^2^: 0.00009 to 0.07). This is supported by other papers where, depending on the species, significant bioaccumulation was not noted [2].

Total measured organ load (muscle + liver) better reflects the overall exposure of the individual animals because heavy metal deposition is dynamic, affecting multiple organs. Species were grouped into epipelagic, mesopelagic and demersal groups for heavy metal-level comparisons. ANOVA with Kruskal–Wallis and Dunn’s correction for multiple comparisons was used to determine statistical differences (*p* < 0.05) in total load concentrations. In addition, within each zone, individual species had total loads compared using a Student *t*-test when 2 species were available (epipelagic zone), and ANOVA with Kruskal–Wallis and Dunn’s correction when 3 species were available (demersal zone). The muscle and liver concentrations of non-essential metals had mean, SDs, and minimum-maximum ranges calculated. A Wilcoxon signed-rank test was used to determine statistical differences between muscle and liver values within each species. Where available, the maximum allowable level or ML food standards obtained from guidelines from EU 2006 [22], Health Canada 2018 [23], and the FAO 1983 [24] was placed in the tables.

It has been suggested that heavy metals can negatively affect fish growth, especially impacting adult weight for length [1]. To verify whether fish sizes (weights) and growth had changed over time and if so were these similar across the various zones were explored. To examine this, historic geographic and age-matched ground fish surveillance trawl morphometric data from 2008/2009 was obtained from Fisheries and Oceans Canada to assess weight for length relationships (LWR) between historical and current data for each species (Groundfish Survey Data, Population Ecology Division (1970–2019), Saint Andrews Biological Station, Saint Andrews New Brunswick, Dartmouth Nova Scotia, Canada, 30 September 2019). Linear regression analysis was performed to determine slopes and intercepts. Then, student *t*-tests using a 95% confidence interval were used to determine statistical differences between historic vs. current data (slopes, intercepts) for each species. Coefficients of determination (r^2^) were also calculated for each LWR using the Pearson’s Correlation test.

## 3. Results

### 3.1. Heavy Metals

#### 3.1.1. Total Measured Organ Load

Antimony was below the detectable levels of the assay (<MDL) in both muscle and liver in all species and was therefore excluded from the tables. When zones were compared (Table 2), total arsenic levels increased with depth across the three water-column zones in both muscle and liver, with the lowest levels seen in the epipelagic species and the highest levels seen in the demersal species. The exception is Acadian redfish, whose levels reflected those seen in the epipelagic zone. Total levels of arsenic were 1.6 times higher in mesopelagic and 3.3 times higher in demersal when compared to epipelagic. The total levels of arsenic in the demersal species were 1.9 times higher than the mesopelagic species. Additionally, the demersal species had the highest levels of nickel, while both the epipelagic and mesopelagic species had no detectable levels of nickel.

**Figure 1:**
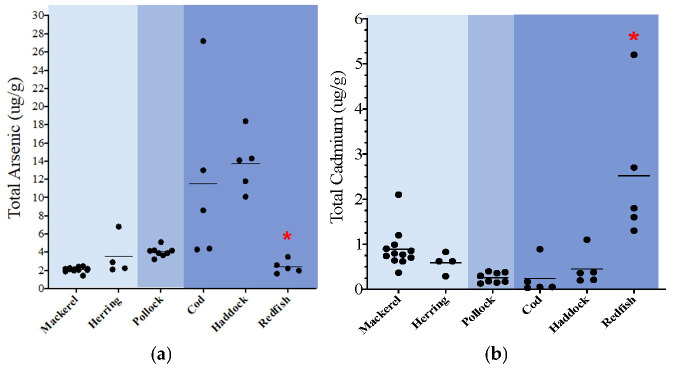

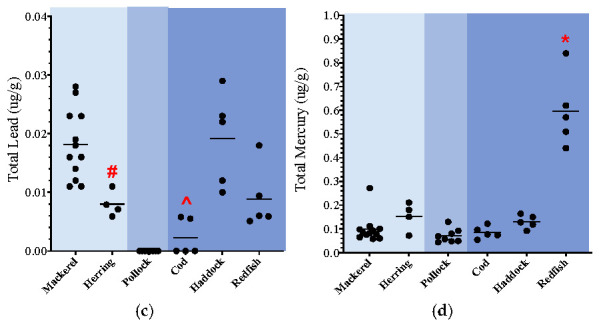
(**a**–**d**): Dot plots of key non-essential metals (ug/g wet weight); total measured organ load = combined muscle and liver levels. * *p* < 0.03 redfish vs. other demersal species; ^ *p* < 0.03 cod vs. other demersal species; ^#^ *p* < 0.03 herring vs. other epipelagic species.

A distinctive trend was seen for cadmium, lead, mercury, and thallium, in which both the epipelagic and demersal species had the highest total levels of metals, while the mesopelagic species had the lowest levels. Specifically, total levels of cadmium were 2.8 times higher in the epipelagic species and 3.5 times higher in the demersal species when compared to mesopelagic. Additionally, total levels of cadmium were 1.2 times higher in demersal species when compared to the epipelagic species. The total levels of lead were <MDL in the mesopelagic species and therefore cannot be directly compared to the epipelagic and demersal species. However, the total levels of lead in the epipelagic species were 2 times higher than what was detected in the demersal species. The total levels of mercury were 1.4 higher in the epipelagic species and 3.5 times higher in the demersal species when compared to mesopelagic. The levels of mercury in demersal were the highest and were 2.5 times higher than the epipelagic species. Finally, the total levels of thallium were <MDL in the mesopelagic species but detected in the epipelagic and demersal species. The levels of thallium were 1.6 times higher in the demersal species when compared to the epipelagic species. When more than one species within a specific zone could be compared, some statistical differences for certain heavy metals were noted between species from the same water-column zone (Figure 1a–d). Specifically, in the epipelagic one, the levels of lead in mackerel were statistically higher than herring, while the levels of thallium in herring were higher than mackerel (Figure 1c). In the demersal zone, Acadian redfish had significantly lower levels of arsenic and higher levels of cadmium and mercury when compared to the other two species (haddock, cod) (Figure 1a,b,d). The levels of nickel and thallium were statistically higher in haddock when compared to cod and redfish, and the lead levels in cod were statistically lower than both haddock and redfish.

#### 3.1.2. Organ Specific (Liver vs. Muscle)

The levels of non-essential metals were compared between muscle and liver (Table 3) within each animal. Overall, heavy metals had significantly higher concentrations in the liver compared to muscle, as indicated in Table 3. Specifically, arsenic and cadmium were statistically higher in the livers of mackerel, pollock, and haddock, while mercury was statistically higher in only mackerel. Redfish livers had statistically higher levels of cadmium and lead, while cod had higher levels of cadmium. The levels of lead were statistically higher in the liver in mackerel and haddock, while nickel was statistically higher in liver in haddock. There were some exceptions, including herring, where no statistical difference was seen for any metal; thallium levels, which were not statistically different in any species; and finally, in 3 species (pollock, cod, and haddock), mercury levels were higher in muscle.

#### 3.1.3. LWR

**Figure 2:**
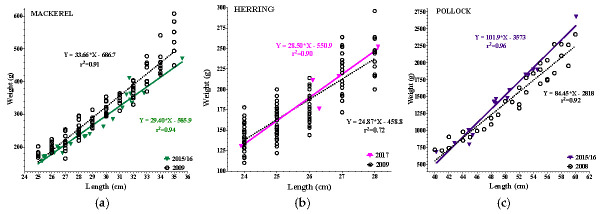

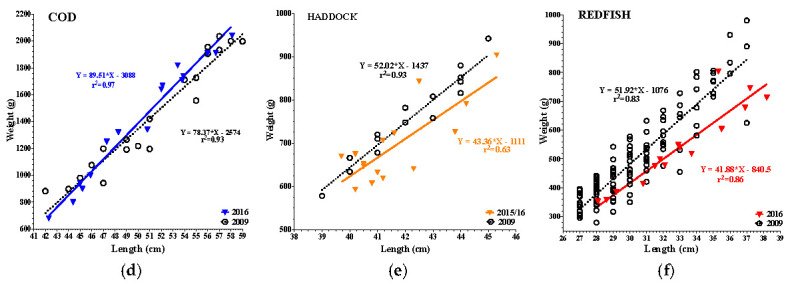
(**a**–**f**): Length–weight relationships (LWR) of 2015–17 vs. 2008/09 species data. Colored lines = current data; black open circles = historic data. (**a**) 2015/16 Mackerel: reduced slope (*p* = 0.05) and intercept (15%). (**b**) 2017 Herring: no LWR reductions. (**c**) 2015/16 Pollock: increased slope (*p* = 0.03) and intercept (23%; *p* = 0.0008). (**d**) 2015/16 Cod: no LWR reductions. (**e**) 2015/16 Haddock: reduced slope (*p* = 0.03) and intercept (25%). (**f**) 2015/16 Redfish: reduced slope (*p* = 0.07) and intercept (24%; *p* < 0.0001).

All species examined had a positive coefficient of determination ranging from r^2^ = 0.63–0.97, while historical data (2008–2009) ranged from r^2^ = 0.83–0.93 (Figure 2a–f). In the epipelagic zone, linear regressions analysis found a 15% reduction in the intercept of mackerel, and a 13% reduction in slope (*p* = 0.05) when compared to 2009. In contrast, herring had no reduction in intercept or slope. In the mesopelagic zone, pollock had a 23% increase in intercept and an 18% increase in slope (*p* = 0.03) compared to 2008. In the demersal zone, haddock had a 25% reduction in intercept and an 18% reduction in slope (*p* = 0.02) compared to 2009. Redfish had a 24% reduction in intercept (*p* = <0.0001) and exhibited a 21% reduction in slope (trend: *p* = 0.07) compared to 2009. Cod had no reduction in either intercept or slope compared to 2009. Overall, reductions in LWR were noted in the epipelagic and demersal zones, whereas the mesopelagic showed a significant increase in LWR.

## 4. Discussion

These unique findings document the levels of non-essential heavy metals in six differing species of fish and confirm that location in the water column does affect non-essential heavy metal accumulation. Since these fish will stray into other zones, identifying specific water-column differences in heavy metals is even more impactful. Importantly, the non-essential metals known to be increased in this region (cadmium, lead, mercury, arsenic) were detected in almost all species. Interestingly, across the narrow age ranges studied, no correlation between absolute age and heavy metal levels was noted.

The specific heavy metal findings of this study include demersal fish having the highest total organ load of arsenic, cadmium, mercury, and nickel. Although gold mining, the major source of arsenic in this region, no longer occurs, deposits in the sediment ensure it persists for decades [25,26,27,28]. Once released from the sediment, arsenic would be found in higher concentrations in zones near the sediment, as demonstrated by the highest levels of accumulation in the demersal. Interestingly, the concentration of arsenic appeared to increase with depth. Specifically, the epipelagic zone had the lowest levels, followed by mesopelagic and then demersal. In support of this concept, a report documenting arsenic levels in flounder, a benthic species, were 1.4 times higher when compared to these demersal species [29]. Other metals such as nickel and thallium are contributed by continued coal combustion and mining [30]. These metals were also found in high concentrations in demersal fish residing near the sediment; however, in contrast to arsenic, these metals were the lowest in the mesopelagic zone.

High levels of cadmium in both the epipelagic and demersal species can be explained by both the storage capacity of cadmium in sediment and its release by climate change, as well as current industrial runoff contributing to high levels of cadmium in surface water. Interestingly, data from a 1997 report on specific trace-element levels in the waters of this region support this finding, as they showed that cadmium levels were 1.4 times higher in the epipelagic and demersal zones when compared to the mesopelagic zone [17]. Epipelagic levels were less than demersal, which could be explained by recent studies that have also classified heavy metal behavior in ocean water based on solubility and their interactions with the surrounding biota. Cadmium is categorized as a “nutrient-like” metal due to phytoplankton in the epipelagic zone consuming this element, leading to a reduction in water concentration could explain the somewhat lower cadmium levels in epipelagic species [18].

Mercury showed a comparable pattern to cadmium with the highest values seen in the epipelagic and demersal zones. Similarly to cadmium, mercury deposits in sediment and acts as a sink, which can account for high levels in demersal species. Additionally, atmospheric deposition into river systems is a major source of mercury in both the BOF and GOM (approx. 78%), potentially leading to the high levels of mercury seen in the epipelagic species residing in surface waters [31].

Levels of lead were undetected in the mesopelagic zone, while the highest concentrations were in the epipelagic and demersal zones. Lead is released into surface waters from industrial runoff, specifically smelting and coal burning, activities which persist on the Atlantic coast, which would contribute to the higher levels seen in the epipelagic species [32]. In addition, higher levels were also found in the demersal species, which could be explained by the additional impact of climate change including increased water temperatures and acidity, which exacerbate release of heavy metals such as lead from sediment [33]. Data from the waters of this region from 1997 also report levels of lead that were three times higher in the epipelagic and demersal zones when compared to mesopelagic [17].

While direct absorption from the water can account for some levels seen, the other major factor which contributes to heavy metals in species is diet, as these metals bio-magnify up the trophic levels. While the six species studied have somewhat similar diets consisting of krill, copepods, and small fish, there are variations which may lead to the species-specific differences which were noted. For example, haddock feed on bottom-dwelling species such as sea stars which reside in and around the sediment, possibly accounting for the higher levels of arsenic and nickel seen, two metals that are found primarily in the sediment [34]. Additionally, krill, a staple in all species’ diets, have been shown to reside in both the epipelagic and demersal zones. In addition, older and therefore larger krill with possibly higher concentrations of metals reside in greater concentrations at depth, perhaps accounting for the higher levels of cadmium and mercury seen in Acadian redfish, whose diet consists primarily of krill [35]. In addition, just how much trophic levels are thought to affect accumulation of heavy metals is becoming more contested, especially within a narrow range [2]. Even so, this alone does not account for the differences seen, as Acadian redfish, a demersal species, has the lowest trophic level of all the fish studied and yet had some of the highest concentrations of heavy metals. Pollock, the mesopelagic species, had the highest trophic level, and some of the lowest concentrations of heavy metals, illustrating that the water-column effects far outweighed all other effects. It is also important to note that there were significant individual animal differences within species seen, which further confirms that this is a complex integrative ecosystem with many sources of heavy metals, either due to their presence in specific water-column zones, foraging behavior, or biological differences [36]. This unique work is the first to report complete nonessential heavy metal profiles in both the muscle and livers of fish. Another report from the Mediterranean Sea selectively examined only the muscle levels of lead, cadmium, and mercury in fish from various water-column depths [37]. Their findings align with our findings, as they did not detect lead and reported demersal fish had double the mercury levels compared to fish in shallower zones [37]. Then, a report from southern China that assessed a few select heavy metals in fish muscle also aligned with our findings that cadmium was highest in the fish of the epipelagic zone [38]. Thus, across vastly differing bodies of water, there is clearly substantial evidence that water-column zones do impact heavy metal levels in fish.

Another major finding of this work is reductions in LWR when compared to historical data seen in mackerel, haddock, and redfish. From these findings, it was shown that the greatest reductions in LWR occurred in fish from both the epipelagic and demersal zones. Interestingly, these fish also had the greatest accumulation of certain non-essential heavy metals, some which have been experimentally linked to growth reductions in fish (arsenic, cadmium). The exception to this finding was cod; however, it has been noted that cod had the shortest age range (2 years) and may also have had their growth previously altered by the cod stock collapse of 1992 [39]. Whether this species had earlier reductions in growth persisting into the 2010s due to the stock collapse is currently unknown and might explain the lack of significant growth changes between 2009 and 2016. Additionally, herring had no reduction in LWR; however, due to fishery closures, sample sizes were limited. Moreover, the data imply that pollock growth has improved, concurrent with pollock having the lowest overall accumulation of non-essential metals, as well as NOAA reporting that this species is not overfished or at risk of overfishing, both possibly contributing to their improved growth [40].

Though not a focus of this work, it bears mentioning that human exposure, particularly to non-essential heavy metals while consuming fish, continues to be of concern. Muscle is the main organ of choice, and the data showed that mercury levels vary between individual fish, with some exceeding the food standards’ maximum allowable levels. This was most notable in select demersal species. Similar observations were noted for the liver, where cadmium also exceeded these standards in all fish, worse in the epipelagic and select demersal species, suggesting caution in the consumption of livers from certain species of fish.

## 5. Conclusions

This study is the first to confirm that the total load of non-essential heavy metals in fish is impacted by the water column in which they are known to reside. Specifically, fish residing in the demersal zone had the highest levels of arsenic and nickel, whereas fish in the epipelagic and demersal zones showed the highest levels of cadmium, lead, mercury, and thallium, and had the greatest reductions in growth. This provides clear evidence for the key role of water-column zones on the environmental impacts of heavy metal contamination in this geographic area. It is possible that with continued anthropogenic pollution and the effects of climate change, concentrations of heavy metals will continue to rise and the role of the water column and proposed stratification of metals cannot be ignored.

## Figures and Tables

**Table 1:** GPS coordinates of all fish catch locations, * their main diets, and trophic levels from FishBase.

Species	Diet *	Trophic Level *	Sample Size	Collection Location
Atlantic Mackerel	Zooplankton, small fish	3.6 ± 0.2 se	n = 25	44°13′43.7″ N 66°15′51.7″ W
Atlantic Herring	Zooplankton, copepods, Small fish	3.4 ± 0.1 se	n = 5	44°6′37.5″ N 66°23′54″ W
Atlantic Pollock	Zooplankton, copepods	4.3 ± 0.4 se	n = 5	44°44′22.6″ N 66°21′47.6″ W
	Small fish		n = 10	43°47′56.9″ N 67°21′46.4″ W
Atlantic Cod	Invertebrates, zooplankton	4.1 ± 0.2 se	n = 3	44°34′7.0″ N 66°40′41.3″ W
	Small fish		n = 13	45°12′52.3″ N 65°48′9.1″ W
Atlantic Haddock	Invertebrates, zooplankton, small fish	4.0 ± 0.1 se	n = 16	44°44′59.8″ N 66°30′49.4″ W
Acadian Redfish	Zooplankton, small fish	3.2 ± 0.2 se	n = 16	44°39′26.9″ N 66°30′16.1″ W

**Table 2:** Total load non-essential heavy metal concentrations in fish occupying the following zones: epipelagic (n = 16; pale blue column), mesopelagic (n = 16; medium blue column), demersal (n = 38; darker blue column). <MDL = method detection limit; for statistical comparisons only, <MDL = 0; SD = standard deviation; min–max = minimum–maximum range; statistical difference (*p* < 0.05): ^a^ vs. epipelagic, ^b^ vs. mesopelagic. (ML)—maximum-allowable-level food standards (EU 2006 [22]; Health Canada 2018 [23]; FAO 1983 [24]).

Total Measured Organ Load µg/g Wet Weight		Epipelagic	Mesopelagic	Demersal
Arsenic (ML = 35)	Mean	2.45	4.29 ^a^	8.18 ^a^
	S.D	1.2	0.93	7.12
	Min-Max	1.42–6.80	3.22–7.20	1.16–27.2
Cadmium (ML = 0.05)	Mean	0.82	0.29 ^a^	1.03 ^b^
	S.D	0.41	0.19	1.23
	Min-Max	0.29–2.10	0.07–0.80	0.02–5.20
Lead (ML = 0.30)	Mean	0.02	0.006 ^a^	0.01
	S.D	0.007	0.007	0.02
	Min-Max	0.006–0.03	<MD–0.02	<MDL–0.09
Mercury (ML = 0.08)	Mean	0.11	0.08	0.28 ^ab^
	S.D	0.06	0.03	0.28
	Min-Max	0.06–0.27	0.04–0.13	0.05–1.42
Nickel	Mean	<MDL	<MDL	0.13 ^ab^
	S.D	<MDL	<MDL	0.29
	Min-Max	<MDL	<MDL	<MDL–1.50
Thallium	Mean	0.0008	<MDL	0.0005
	S.D	0.001	<MDL	0.001
	Min-Max	<MDL–0.004	<MDL	<MDL–0.006

**Table 3:** (**a**) Muscle levels of non-essential heavy metals concentrations in mackerel (n = 12), herring (n = 4), pollock (n = 16), cod (n = 13), haddock (n = 9), redfish (n = 16). <MDL = method detection limit; for statistical comparisons only, <MDL = 0; SD = standard deviation; min–max = minimum–maximum range; lead and nickel were <MDL in muscle and not included. Muscle values were compared to liver values with statistics reported in Table 3. (ML = maximum-allowable-level food standards (EU 2006 [22]; Health Canada 2018 [23]; FAO 1983 [24]). (**b**) Liver levels of non-essential heavy metals concentrations in mackerel (n = 12), herring (n = 4), pollock (n = 16), cod (n = 13), haddock (n = 9), redfish (n = 16). <MDL = method detection limit; for statistical comparisons only, <MDL = 0; SD = standard deviation; min–max = minimum–maximum range. *p* < 0.008: ^*^ vs. muscle (found in Table 3). (ML = maximum-allowable-level food standards (EU 2006 [22]; Health Canada 2018 [23]; FAO 1983 [24]).

(a)							
Muscle (µg/g Wet Weight)							
		Epipelagic		Mesopelagic	Demersal		
		Mackerel	Herring	Pollock	Cod	Haddock	Redfish
Arsenic (ML = 35)	Mean	0.57	1.16	1.13	5.83	4.07	1.26
	SD	0.18	0.58	0.60	5.37	2.15	1.08
	Min–Max	0.37–0.95	0.70–2.00	0.44–2.9	1.9–18.0	1.4–6.9	0.35–4.30
Cadmium (ML = 0.05)	Mean	0.003	<MDL	0.001	<MDL	0.0004	0.0003
	SD	0.005	<MDL	0.005	<MDL	0.001	0.001
	Min–Max	<MDL–0.01	<MDL	<MDL–0.02	<MDL	<MDL–0.004	<MDL–0.005
Mercury (ML = 0.08)	Mean	0.03	0.08	0.07	0.06	0.08	0.27
	SD	0.02	0.04	0.03	0.02	0.01	0.16
	Min–Max	0.02–0.08	0.04–0.13	0.04–0.12	0.03–0.10	0.06–0.12	0.08–0.74
Thallium	Mean	<MDL	0.003	<MDL	<MDL	<MDL	<MDL
	SD	<MDL	0.001	<MDL	<MDL	<MDL	<MDL
	Min–Max	<MDL	0.002–0.004	<MDL	<MDL	<MDL	<MDL
(**b**)							
**Liver (µg/g Wet Weight)**							
		**Epipelagic**		**Mesopelagic**	**Demersal**		
		**Mackerel**	**Herring**	**Pollock**	**Cod**	**Haddock**	**Redfish**
Arsenic (ML = 35)	Mean	1.5 *	2.35	3.16 *	4.85	10.6 *	1.2
	SD	0.36	1.64	0.6	2.20	4.92	0.28
	Min–Max	0.56–2.0	1.40–4.80	2.0–4.30	2.3–9.20	7.3–23.0	0.88–1.70
Cadmium (ML = 0.05)	Mean	0.89 *	0.59	0.29 *	0.21 *	0.48 *	2.01 *
	SD	0.43	0.22	0.19	0.33	0.31	1.35
	Min–Max	0.37–2.1	0.29–0.83	0.07–0.78	0.02–1.00	0.20–1.10	0.31–5.2
Lead (ML = 0.3)	Mean	0.02 *	0.008	0.003	0.001	0.03 *	0.01 *
	SD	0.006	0.002	0.004	0.003	0.02	0.005
	Min–Max	0.01–0.03	0.006–0.01	<MDL–0.01	<MDL–0.006	0.01–0.09	<MDL–0.02
Mercury (ML = 0.08)	Mean	0.07 *	0.07	0.01 *	0.04 *	0.05 *	0.25
	SD	0.04	0.03	0.007	0.007	0.02	0.17
	Min–Max	0.03–0.19	0.04–0.11	0.004–0.03	0.03–0.05	0.03–0.08	0.06–0.68
Nickel	Mean	<MDL	<MDL	<MDL	<MDL	0.54 *	0.02
	SD	<MDL	<MDL	<MDL	<MDL	0.37	0.03
	Min–Max	<MDL	<MDL	<MDL	<MDL	0.30–1.50	<MDL–0.11
Thallium	Mean	<MDL	0.003	<MDL	<MDL	0.002	<MDL
	SD	<MDL	0.001	<MDL	<MDL	0.002	<MDL
	Min–Max	<MDL	0.002–0.004	<MDL	<MDL	<MDL–0.006	<MDL

## Data Availability

The original contributions presented in this study are included in the article/Supplementary Material. Further inquiries can be directed to the corresponding author(s).

## Supplementary Materials

The following supporting information can be downloaded at https://www.mdpi.com/article/10.3390/toxics13060419/s1, Table S1: MDLs (µg/g wet weight) provided by the Animal Health Laboratory at the University of Guelph.

## Abbreviations

The following abbreviations are used in this manuscript:

BOF	Bay of Fundy
GOM	Gulf of Maine
LOQ	Limit of Quantification
LWR	Weight for length relationship
MDL	Method Detection Limit
SD	Standard Deviation
