# Supplementary material for: Water-Column Zone Impacts Non-Essential Heavy Metal Accumulation in Fish Occupying Different Zones
Source: Toxics. 2025 May 22;13(6):419. doi: 10.3390/toxics13060419 (PMC12197239; doi:10.3390/toxics13060419)
Supplement: Supplementary file 1 [file toxics-13-00419-s001.zip › toxics-3586874-supplementary.pdf]

Table S1. MDLs (µg/g wet weight) provided by the Animal Health Laboratory at the University of Guelph.

Element	MDL
Antimony	0.006
Arsenic	0.004
Cadmium	0.004
Lead	0.005
Mercury	0.001
Nickel	0.11
Thallium	0.002
